# Rationale and design of the validation of bladder health instrument for evaluation in women (VIEW) protocol

**DOI:** 10.1186/s12905-020-01136-w

**Published:** 2021-01-07

**Authors:** Emily S. Lukacz, Melissa L. Constantine, Lisa Kane Low, Jerry L. Lowder, Alayne D. Markland, Elizabeth R. Mueller, Diane K. Newman, Leslie M. Rickey, Todd Rockwood, Kyle Rudser, for the Prevention of Lower Urinary Tract Symptoms (PLUS) Research Consortium

**Affiliations:** 1 Division of Female Pelvic Medicine and Reconstructive Surgery, University of California San Diego, San Diego, CA USA; 2 Division of Biostatistics, University of Minnesota, Minneapolis, MN USA; 3 Practice and Professional Graduate Programs, School of Nursing, University of Michigan, Ann Arbor, MI USA; 4 Division of Female Pelvic Medicine and Reconstructive Surgery, Washington University in St. Louis, St. Louis, MO USA; 5 Division of Gerontology, Geriatrics, and Palliative Care, Department of Medicine, University of Alabama At Birmingham, Birmingham, AL USA; 6 Department of Veterans Affairs, Birmingham/Atlanta Geriatric Research, Education, and Clinical Center (GRECC), Birmingham, AL USA; 7 Division of Female Pelvic Medicine and Reconstructive Surgery, Loyola University Medical Center, Loyola University Chicago, Maywood, IL USA; 8 Division of Urology, Department of Surgery, University of Pennsylvania Perelman School of Medicine, Philadelphia, PA USA; 9 Departments of Urology and Obstetrics, Gynecology and Reproductive Sciences, Yale University, New Haven, CT USA; 10 Division of Health Policy and Management, University of Minnesota, Minneapolis, MN USA; 11 National Institutes of Health, Bethesda, MD USA; 12 Department of Obstetrics, Gynecology and Reproductive Sciences, UC San Diego Health, 9500 Gilman Dr. #0971, La Jolla, CA 92093 USA

**Keywords:** Bladder, Health, Instrument, Measurement, Questionnaire, Scale, Validation

## Abstract

**Background:**

Bladder health is an understudied state and difficult to measure due to lack of valid and reliable instruments. While condition specific questionnaires assess presence, severity and degree of bother from lower urinary tract symptoms, the absence of symptoms is insufficient to assume bladder health. This study describes the methodology used to validate a novel bladder health instrument to measure the spectrum of bladder health from very healthy to very unhealthy in population based and clinical research.

**Methods:**

Three samples of women are being recruited: a sample from a nationally representative general population and two locally recruited clinical center samples—women with a targeted range of symptom severity and type, and a postpartum group. The general population sample includes 694 women, 18 years or older, randomly selected from a US Postal delivery sequence file. Participants are randomly assigned to electronic or paper versions of the bladder health instrument along with a battery of criterion questionnaires and a demographic survey; followed by a retest or a two-day voiding symptom diary. A total of 354 women around 7 clinical centers are being recruited across a spectrum of self-reported symptoms and randomized to mode of completion. They complete the two-day voiding symptom diary as well as a one-day frequency volume diary prior to an in-person evaluation with a standardized cough stress test, non-invasive urine flowmetry, chemical urine analysis and post void residual measurement. Independent judge ratings of bladder health are obtained by interview with a qualified health care provider. A total of 154 postpartum women recruited around 6 of the centers are completing similar assessments within 6–12 weeks postpartum. Dimensional validity will be evaluated using factor analysis and principal components analysis with varimax rotation, and internal consistency with Cronbach’s alpha. Criterion validity will be assessed using multitrait-multimethod matrix including correlations across multiple data sources and multiple types of measures.

**Discussion:**

We aim to validate a bladder health instrument to measure the degree of bladder health within the general population and among women (including postpartum) recruited from local clinical centers.

*Trial registration* NCT04016298 Posted July 11, 2019 (https://www.clinicaltrials.gov/ct2/show/NCT04016298?cond=bladder+health&draw=2&rank=1).

## Background

Methods to promote bladder health and prevent lower urinary tract symptoms (LUTS) are poorly understood and strategies for decreasing risk of LUTS across the lifespan of girls and women are lacking. In order to better understand the impact of bladder health promotion and LUTS prevention strategies, valid measures for assessing bladder health are urgently needed. Historically, and like most medical conditions, bladder health has been assumed through the presence or absence of symptoms. Existing instruments have been primarily designed to measure LUTS and assess impact of interventions in clinical populations or the burden of disease in epidemiologic studies. While reports of “normative data” and “healthy function” have been published, these are limited by the fact that the study populations are typically poorly characterized with respect to bladder health and described as “normal”, “healthy”, or “asymptomatic” based purely on the absence of LUTS [1–3]. Additionally, while there are numerous terms and definitions to describe bladder disease, a definition and measure of bladder health did not previously exist. Thus, a true estimate of women’s bladder health in the general population is currently not attainable.

The Prevention of Lower Urinary Tract Symptoms (PLUS) Research Consortium was established in 2015 with the primary charge of identifying and promoting bladder health (BH) [4]. By employing a rigorous prevention-based research agenda, the ultimate goal is to understand how to promote and preserve bladder health and prevent LUTS over the life course of girls and women [5, 6]. Essential toward this aim is an assessment of the distribution of bladder health in the general population. An initial step in the process was to establish a formal definition of bladder health for use in the development of a valid measure [7]. Consistent with the World Health Organization’s definition of health [8]. The PLUS Consortium conceptualizes bladder health as *“*a complete state of physical, mental, and social well-being related to bladder function, and not merely the absence of lower urinary tract symptoms (LUTS). Healthy bladder function permits daily activities, adapts to short term physical or environmental stressors, and allows optimal well-being (e.g., travel, exercise, social, occupational or other activities)” [9].

Measuring degrees of bladder health in a variety of settings requires a valid instrument, ideally self-administered, that can capture a spectrum from very healthy to very unhealthy. The PLUS bladder health item pool was generated using expert opinion, review and adaptation of existing instruments, and drafting of novel items to assess the elements of our conceptual model of bladder health. This content valid item pool is referred to as the Bladder Health Instrument (BHI), with the aim of further development and evaluation to result in a Bladder Health Scale (BHS). The BHI items and language were further refined by cognitive evaluation with community dwelling women and review of focus group data, as the initial step in scale development [10]. The primary objective of this paper is to describe the methodology employed by the PLUS Research Consortium for scale development and further scale evaluation, including: (1) assess the reliability and validity of the PLUS-BHI for measurement of bladder health among adult women and (2) evaluate effects of mode of administration of the PLUS-BHI in order to create a scale that is mode agnostic. This will support its use in future research in population-based national studies and future prevention trials.

## Methods/design

### Overview

The Validation of Bladder Health Instrument for Evaluation in Women (VIEW) study is an IRB approved (ADVARRA #Pro00032238), multicenter study funded by the National Institute of Diabetes and Digestive and Kidney Diseases (NIDDK) (NCT04016298), designed to test a questionnaire that can measure the degree of bladder health in epidemiologic research conducted across different populations and recruitment strategies as well as for use in local community intervention studies. The VIEW study builds upon the internal validation of the item pool for the instrument by evaluating dimensional validity and internal reliability and further establishes external validity. The goal is to create a Bladder Health Scale (PLUS-BHS) to establish basic inferential validity and to draw valid inferences about the distribution of bladder health in women in the general population and also in clinical research. Therefore, it is essential that the populations represented in the validation samples have some equivalence to the target populations the PLUS-BHI is intended to be used in [11]. The VIEW study protocol includes three population samples: a sample from a nationally representative general population and two locally recruited clinical center samples—women with a targeted range of symptom severity and type, and a postpartum group. Postpartum women are a focal population identified for future research due to the known high risk of developing LUTS in the peripartum period and evidence for successful LUTS prevention strategies [12, 13]. Recalling peripartum LUTS is inaccurate when assessed remote from the delivery, resulting in high recall bias and measurement error [14]. Adequate representation of this important population is critical to establish confidence that the PLUS-BHS will allow valid inference to be made by future studies of this population.

Effect of mode administration is a well-documented phenomenon in survey research [15, 16]. In addition to context-based effects associated with mode of survey administration [17–20], other potential biases or sources of error associated with mode of administration are unknown. While a computer mode may increase efficiency of questionnaire completion, it also may produce measurement error. Thus, use of a computer may alter the distribution of PLUS-BHS [21, 22]. We intentionally include both paper and electronic modes in VIEW in order for the PLUS-BHS construction resulting from this validation process to be mode agnostic.

Measuring degrees of bladder health in a variety of settings requires a valid instrument, ideally self-administered, that can capture a spectrum from very healthy to very unhealthy. The PLUS Bladder Health Instrument (PLUS-BHI) item pool was generated using expert opinion, review and adaptation of existing instruments, and drafting of novel items to assess the elements of our conceptual model of bladder health. The items and language were refined by cognitive evaluation with community dwelling women and review of focus group data [10].

### Study populations

Inclusion criteria for the general and clinical center populations are outlined in Table 1. In general, non-pregnant, ambulatory women 18 yrs of age or older are targeted for enrollment.

**Table 1:** Eligibility criteria for the general, clinical, and post-partum populations

General population	Clinical center population	Postpartum population*
*Inclusion criteria*		
Community dwelling Age ≥ 18 years old Female sex assigned at birth Fluent in written and spoken English Able to read and provide informed consent	Same inclusion criteria as general population *Additional criteria* Stand independently without human assist Able to stand and toilet independently Willing to complete PLUS-BHI validation survey and 2-day Bladder Health Symptom diary and 1-day Bladder Health Frequency-Volume diary prior to in-person clinical evaluation Willing to commit to an in-person evaluation within 8 weeks of enrollment	Same inclusion criteria as general and clinical populations *Additional criteria* Pregnant in 3rd trimester or less than 12 weeks postpartum Available and willing to come for an in-person evaluation within 8–12 weeks postpartum (may be enrolled prior to delivery)
*Exclusion criteria*		
	*Additional criteria* Pregnant at the time of data collection or within 12 weeks postpartum Diagnosis or history of bladder cancer, kidney transplant, pelvic radiation, or currently getting dialysis Current participation in a research study about bladder	Same exclusion criteria as the Clinical Population with the exception of being pregnant if enrolled in the 3rd trimester*

^*^Postpartum population eligibility is independent of mode of delivery (spontaneous vaginal, operative vaginal, and cesarean deliveries)

#### General population

The US Postal delivery sequence file (DSF), an address-based sample frame, is used to draw a random nationally representative sample. The DSF is the gold standard for address based sampling of US households with low coverage error [23]. In order to have geographic representation in terms of region of the country and across urban and rural continuum, a stratified sampling strategy was implemented with three nested sampling unit levels: primary sampling unit (PSU), secondary sampling unit 1 (SSU1) and a secondary sampling unit 2 (SSU2). The primary sampling unit mimics the PSU groupings used by the National Health And Nutrition Examination Survey (NHANES), [24] with states aggregated into one of five strata based on state health indicators. Each PSU is further divided between 0 and 2 times based on general geographic proximity (e.g., East, Midwest and West) resulting in a total of 11 SSU1 strata across the 5 PSUs (Table 2).

**Table 2:** General population sampling SSU1 state grouping

SSU1 state grouping	States included
Group 1: PSU 1, West	HI, UT, WA
Group 2: PSU 1, Midwest	IA, MN, ND
Group 3: PSU 1, East	CT, MA, NH, NJ, NY, VT, RI
Group 4: PSU 2, CA	CA
Group 5: PSU 3, West	AK, AZ, CO, ID, MT, NM, OR, WY
Group 6: PSU 3, Midwest	IL, KS, ME, NE, SD, WI
Group 7: PSU 3, South	FL, VA
Group 8: PSU 4, TX	TX
Group 9: PSU 4, East	DE, IN, MD*, MI, OH, PA
Group 10: PSU 5, Western South	AR, LA, MO, NV, OK
Group 11: PSU 5, Eastern South	AL, KY, GA, MS, NC, SC, TN, WV

^*^DC counted in MD

PSU, Primary sampling unit; SSU, Secondary sampling unit

Each of the 11 SSU1 were further stratified as SSU2 according to the rural urban continuum codes (RUCC). These RUCC were categorized into four groups: urban (RUCC 1 and 2), suburban (RUCC 3, 4 and 6), rural city (RUCC 5 and 7), and rural (RUCC 8-9). A total frame of 6000 households (for a target of approximately 694 completed validation surveys) is randomly selected across sampling strata. Sampling is proportional across SSU1 state groupings, and equal across the 4 RUCC groups for a total of 44 sampling strata (Table 2).

#### Clinical center populations

The general clinical center population of women are being recruited from the community and medical practices surrounding the PLUS clinical research centers: Loyola University Chicago, University of Alabama at Birmingham, University of California San Diego, University of Michigan, University of Pennsylvania, Washington University in St Louis, and Yale University. Potential participants are screened on inclusion/exclusion criteria (Table 1) and purposive sampling conducted with the intention of representing the spectrum of bladder health and LUTS. To accomplish this, a modified version of the Patient Perception of Bladder Condition (PPBC) questionnaire is used to categorize participants into one of four equal size groupings [25]. Using the question “Which of the following statements best describes any problems you may have with peeing or your bladder?”: women are screened into healthy with no reported problems (PPBC response = 1), mild bladder problems (PPBC response = 2 or 3), moderate bladder problems (PPBC response = 4), or severe bladder problems (PPBC response = 5 or 6). When a quota is full (i.e., severe), individuals are screened out (Table 3).

**Table 3:** VIEW target enrollment of clinical center population bladder symptom strata

Symptom strata	Self-report of LUTS-PPBC				
	Healthy	Mild	Moderate	Severe	Total
Frequency	90	15	15	15	45+
Incontinence		15	15	15	45+
Urgency		15	15	15	45+
Pain/discomfort		15	15	15	45+
Peeing/flow		15	15	15	45+
Urinary tract infection		15	15	15	45+
Total	90	88	88	88	354

LUTS, Lower urinary tract symptoms; PPBC, Patient perception of bladder condition: The four categories of Healthy to Severe map to PPBC responses to the question “Which of the following statements best describes any problems you may have with peeing or your bladder?”: healthy with no reported problems (PPBC response = 1), mild bladder problems (PPBC response = 2 or 3), moderate bladder problems (PPBC response = 4), or severe bladder problems (PPBC response = 5 or 6)

In order to ensure a sufficient representation across a spectrum of LUTS in the sample sites, a second level of screening is also employed using screening items assessing presence or absence of urinary frequency, urinary urgency, urinary incontinence, urinary pain, and difficulty voiding. Additionally, each center is required to enroll a minimum of 5 participants into each of the 4 age strata: 18–25, 26–45, 46–65, and 65+ years of age. For the postpartum population, women are recruited from 6 of the clinical centers during late pregnancy or around the time of delivery with the inclusion and exclusion criteria noted in Table 1. The timing of assessment of bladder health in the postpartum period is circumscribed to 6–12 weeks after delivery. Recruitment from the obstetric population is occurring simultaneously to those recruited from the clinical center population described above. Due to the limited age range and limited range of bladder health in the postpartum population, there are no specific age targets, or self-report of LUTS.

### Survey mode

The intended use of the validated PLUS-BHS is a self-administered questionnaire for women in general population research as well as in clinical research. Given the differing sampling approaches typically used for these different types of research, our goal is to ensure the PLUS-BHS is valid for use across two likely modes of administration: paper and pencil instrument (PAPI) and computer assisted instrument (CASI).

### Study administration

#### General population

**Fig. 1:**
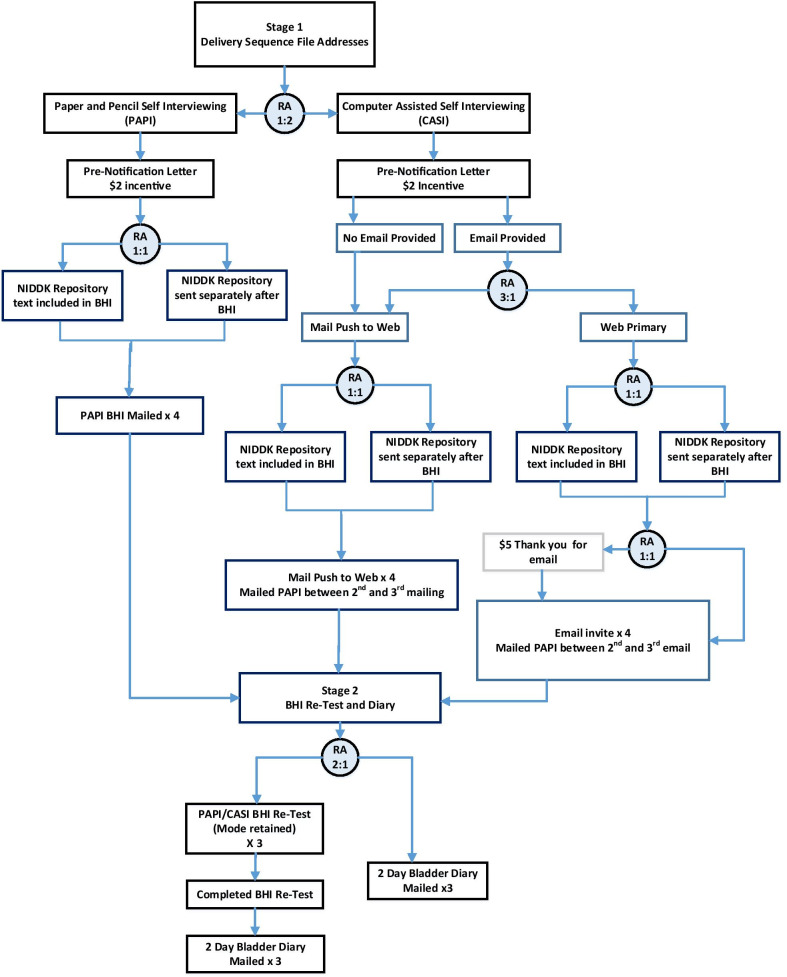
General population study flow. BHI, Bladder Health Instrument; CASI, Computer Assisted Self Interview; PAPI, Paper and Pencil self-Interview; RA, Random Assignment

The study flow for the general population cohort is outlined in Fig. 1. All households in the DSF sample frame are mailed a pre-notification letter including a $2.00 bill and a tri-fold color brochure describing the study, with a request for participation by the female in the household, age 18 or over with the most recent birthday. Households are randomized 1:2 to PAPI or CASI. The imbalanced randomization reflects an expected lower response rate from CASI [23]. For those randomized to PAPI, the first PLUS-BHI packet mailing includes $10 as incentive and a stamped envelope addressed to the Scientific and Data Coordinating Center (SDCC). Non-respondents are sent replacement packets up to 4 times. Those randomized to CASI are asked to provide a valid email address using the provided postage paid return envelope. Respondents who do not provide an email are necessarily assigned to the push-to-web group, wherein future paper mailings include an electronic link for online survey completion. Households who do provide an email address are randomized to either the push-to-web group or to receive an email with a link to the survey (referred to as the web primary group). This second randomization allows a full comparison of respondents who fully elect to respond via electronic mode to those who do not. Those in the web primary group are additionally randomized 1:1 to receive $5 thank you for providing an e-mail address. A total of four follow-up packets are mailed to non-respondent households wherein the final contact includes a paper version of the survey.

Respondents, regardless of initial mode assignment, who fully complete the validation survey packet are subsequently re-contacted within 2–4 weeks and randomized 2:1 to complete either (a) a retest of the PLUS-BHI in the same mode (PAPI or CASI) followed by a 2-day bladder symptom diary (Additional file 1) or (b) to only complete a 2-day bladder symptom diary. The retest version of the PLUS-BHI includes 2 Guyatt transition rating items [26] to identify changes to a person’s health, which serve as an anchor for stability in the retest reliability analysis, as well as the PLUS-BHI items. The 2-day bladder symptom diary is mailed to the respondent independent of mode of PLUS-BHI completion. Up to 4 contact attempts are made for completion of the retest version of the PLUS-BHI and up to 3 contact attempts for completion of the 2-day bladder symptom diary. Respondents receive $10 compensation for completing each of the re-test and/or 2-day bladder symptom diary.

The NIDDK has an expectation of grant award recipients to support data sharing, facilitated by their data repository. To assess the impact of language related to data sharing in the consent process, each address is randomly assigned (1:1) to have the request for permission to share anonymized data with the NIDDK repository either at the end of the PLUS-BHI validation packet or to receive the request separately (e-mail for CASI, separate letter for PAPI). This allows for evaluation of potential threat bias influence on completion of the survey altogether and also the degree of endorsement separate from completion of the survey [23].

#### Clinical center populations

**Fig. 2:**
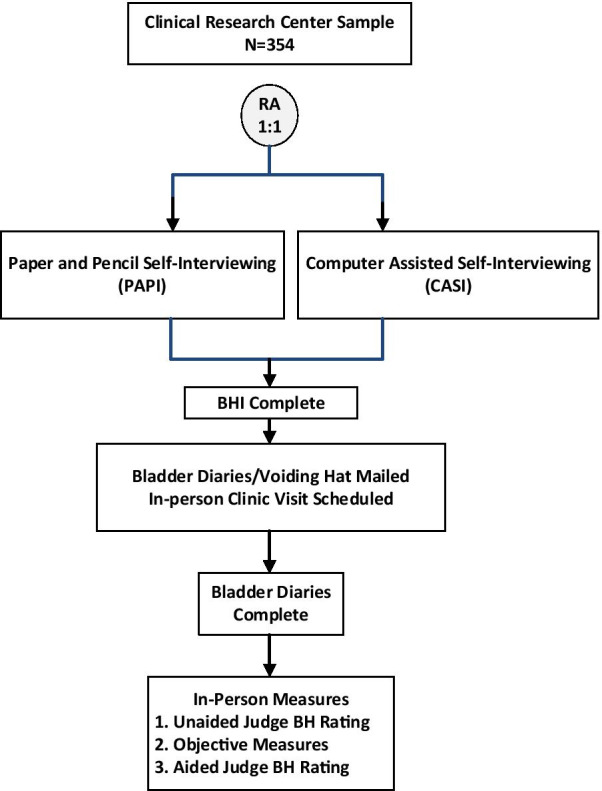
Clinical center sample study flow. BH, Bladder Health; BHI, Bladder Health Instrument; CASI, Computer Assisted Self Interview; PAPI, Paper and Pencil self-Interview; RA, Random Assignment. *Note*: Postpartum sample N = 154 and are not randomized to BHI mode of administration

The study flow for the general population cohort is outlined in Fig. 2. Once eligibility criteria are established (Table 1), verbal consent is obtained; and participants are enrolled and randomized to PAPI vs. CASI mode 1:2 in blocks of 3, stratified by center. Those who do not have email capability are offered participation in the PAPI arm. Those assigned to PAPI mode are mailed the same survey packet described for the general population. Those participants assigned to the CASI version are emailed a unique link to complete the surveys and PLUS-BHI validation material online, which are captured directly via REDCap [27]. Non-respondents are mailed or emailed replacement packets up to three times every 15 days.

Participants who complete the initial materials are contacted and scheduled for an in-person clinical evaluation. Upon scheduling they are mailed a decorative box containing a copy of the written consent form, the 2-day bladder symptom diary (Additional file 1), a 1-day frequency-volume bladder dairy (Additional file 2), a voiding hat to measure urine volumes, and detailed instructions for completion of all material. Participants are contacted at least 4 days prior to the clinical evaluation to ensure completion of diaries prior to the visit.

On the day of the in-person evaluation, participants proceed with written informed consent, and bladder diaries are reviewed to confirm completion. Participants undergo an interview with a bladder health judge followed by clinical testing procedures to obtain the objective measures of bladder function outlined below. Participants receive prorated compensation up to $100 for completion of the study.

Women recruited for the postpartum population are allowed to select their preferred mode of administration rather than be randomized given the additional demands of completing study procedures for new mothers. The survey packet is sent to be completed within the 6–12 weeks postpartum window. Otherwise the steps are identical to the general clinical center population with respect to the judge assessments and in-person clinical evaluation conducted.

### Measures

Demographics items include both standard demographics as well as items to assess gender identification and sexual orientation. Items to assess general medical history and history of pelvic floor disorders are included. The survey packet also includes items to assess current LUTS and validated measures that serve as external criterion in establishing external validity. Details of the criterion instruments are further described below (Table 4). Dependent on branching, the number of items a respondent is presented ranges from 63 to 90 items. The total PAPI survey length, inclusive of all self-report measures, is 49 pages.

**Table 4:** External criterion measures for validation assessment

Measure	Data source	Data method	Validation type
*Same source, same method (self-reported measures)*			
PLUS-BHI	Self	Survey	–
MOS-SF 36*	Self	Survey	Convergent
KHQ	Self	Survey	Convergent
UDI-6	Self	Survey	Convergent
POPDI	Self	Survey	Divergent
CRADI	Self	Survey	Divergent
BFLUTS	Self	Survey	Convergent
*Same source, different method*			
Bladder diaries: 1-day 2-day	Self	Activity log	Convergent
*Different source, different method*			
Judge BH rating: Aided Unaided	Other/expert	Qualitative	Convergent Divergent
*Different independent source, different method (objective measures)*			
PTT	Other/technical	Volume calculation	Convergent Divergent
Uroflowmetry	Voiding dynamics	Rate calculation	Convergent Divergent
PVR volume	Imaging	Volume calculation	Convergent Divergent
Urinalysis	Dipstick/biologic	Standardized lab Analysis	Convergent Divergent

^*^Ten selected items from the Medical Outcome Study Short Form Health Survey (MOS SF-36)

KHQ, King’s Health Questionnaire; UDI, Urogenital Distress Inventory; BFLUTS, Bristol Female Lower Urinary Tract; PFDI-20, Pelvic Floor Distress Inventory; CRADI, colorectal and anal distress inventory; POPDI, pelvic organ prolapse distress inventory; PPT, Paper Towel Test for stress incontinence; PVR, post-void residual volume measurement by bladder scan

The intended use of a measure dictates the level of evidence required for inferential validity of a measure [28–30]. For this work, we are employing Campbell and Fiske’s multitrait-multimethod (MTMM) matrix to evaluate hierarchical and cumulative levels of evidence for convergent and discriminant validation of PLUS-BHS score [31]. External variables that rely on multiple and independent sources and methods of data collection in comparison to dimensions of the emergent PLUS-BHS will provide a high level of evidence to support inference of PLUS-BHS score as valid for research in the general population as well as clinical research. The external measures employed by this study were identified to satisfy the requirements of multiple sources and multiple methods: the referent method is self-report survey and the referent source is study participant.

#### Same source/same method measures

The PLUS consortium reviewed the quality and strength of evidence of available validated instruments that will provide measures of LUTS to serve as same source same method external criteria. Multiple instruments were assessed based on their potential ability to serve as a comparator to the bladder dimensions and domains of the bladder health model defined by the PLUS consortium [10]. Table 4 describes the instruments selected for use as criterion measures in the VIEW study. These include ten general health items selected from the Medical Outcome Study (MOS) Short Form-36 to evaluate association with the general bladder related health dimension of bladder health [32]. The King’s Health Questionnaire (KHQ) [33] and Urogenital Distress Inventory (UDI) short form [34] recommended as Grade A instruments for use in LUTS research were included [35]. However, the consortium determined that the KHQ and UDI may have insufficient items for evaluation of emptying function, thus the Bristol Female Lower Urinary Tract Symptoms (BFLUTS 3 voiding items (V1–V3)) [36] were included. Finally, the Pelvic Floor Distress Inventory (PFDI-20) [37], which includes the colorectal (CRADI) and prolapse (POPDI) subscales along with the UDI, were included with the expectation they will serve to evaluate divergence [37]. These items were administered to all three cohorts (general population, general clinical and postpartum clinical center cohorts) in the same mode as assigned for the PLUS-BHI completion.

#### Same source/different method

The 2-day bladder symptom diary was developed by the PLUS consortium as an expanded version of a voiding record assessing storage symptoms (frequency, continence, sensation of urge and pain) and emptying symptoms (initiation, flow, efficacy, relief of urge sensation and pain) along with fluid intake and absorbent product usage over 2 days (Additional file 1). It is completed by a subset of women recruited from the general population and by all clinical center recruited women. The 1-day frequency/volume diary (Additional file 2) is being used to collect voided volume, frequency of urination, presence and severity of urinary leakage episodes, pad usage and fluid intake, and is completed only by clinical center recruited women.

#### Different source/different method

The inferential utility and validity of the PLUS-BHS will in part be determined by its comparability, as a measure of self-reported bladder health to what are deemed standard clinical judgements of bladder health. In order to capture this clinical judgement or rating of bladder health, each clinical research center has designated a variety of bladder health raters (a.k.a. judges). These judges are health care providers with a point of view which makes their clinical observation of bladder health a viable standard which can be used as a criterion. All women recruited for the clinical center populations meet with a judge who provides an overall bladder health assessment. For purposes of this study, a judge is considered a health care provider who is able to respond to basic questions or statements about the bladder from a participant. Operationally, we consider any provider (e.g., nurse practitioner, physician assistant, nurse midwife, physician, or surgeon) in family practice, internal medicine, geriatrics, obstetrics-gynecology, midwifery, urology, with or without subspecialty training in female urologic conditions. This design is expected to provide heterogeneity among judges and ensure generalizability that would not be possible using standardized evaluation procedures or only bladder specialists. This study also includes a balanced proportion of judges who are and are not engaged in the PLUS consortium, in order to minimize biases related to having heightened awareness of bladder health as a PLUS Consortium member.

Following the in-person interview with the participant, the judge provides two initial unaided ratings of bladder health: a circumstance or context adjusted, “relative” rating of bladder health as well as an “absolute” rating of bladder health (Additional file 3). No specific script or checklist for this rating is mandated; each judge may use whatever method they generally employ in their practice to assess their patients relative to bladder health. Along with each numerical rating (0–10), the judge is asked to provide the 3 most important factors that contribute to the rating. Following the completion of these initial ratings, the judge is provided with the participants 1-day frequency-volume bladder diary as well as the clinical test results (described below) and any other data available from standard of care clinical practice gathered during the interview process. This material is used by the judge to inform a second set of “relative” and “absolute” bladder health ratings along with the description of the 3 most important factors that contributed to each rating. Where practically feasible, the participant-judge interaction is scheduled to occur prior to the administration of clinical tests in order to minimize potential for test effect influence on participant’s interaction with the judge.

#### Different independent source/different method

Clinical tests completed during the in-person visit are measures that qualify as independent and objective data sources that provide evidence for a higher level of inferential validity. These tests are outlined in Table 4. In-person clinical measures included a quantified standing (provocative) paper towel test (PTT) for stress incontinence [38]; a non-instrumented, seated uroflowmetry with a minimum pre-void volume measurement of 150 mL assessed by bladder scan to determine volume, speed of flow, pattern and length of voiding; a non-invasive post-void residual (PVR) volume measurement by bladder scan; and a chemical urine analysis (a.k.a. dipstick) recording pH, specific gravity, blood, glucose, protein, leukocyte and nitrites to asses hydration status, infection and hematuria.

### Analysis plan

Item distributions will be evaluated for floor and ceiling effects. Items that exhibit greater than 85% response in the tail of the distribution will be removed. Item non-response will be evaluated with items demonstrating greater than 20% non-response considered for removal. Test–retest reliability will be evaluated for all items comparing item response on initial PLUS-BHI completion to item response on retest version of PLUS-BHI using Bland–Altman with 95% interval agreement threshold will be used for comparison of continuous variables (including categorical variables with greater than 6-point response options), and chi-square used for comparison of categorical variables, with Fisher’s exact used as needed.

A comparison of responses by mode (PAPI vs. CASI) will be made both between and within responses from the general population sample and the clinical samples separately. Items will be evaluated using Tukey’s HSD or chi-square (Fishers as needed). Items demonstrating significant differences across mode of administration or across sample will be considered for removal.

Internal dimensional validity will be identified and evaluated using factor analytic approaches. Two investigators utilizing multiple methods principal component analysis and factor analysis and rotation methods (orthogonal/oblique) will work independently and periodically compare and discuss results. Internal consistency of item groupings will be evaluated with Cronbach’s alpha. Item groupings with alpha > 0.9 will be further evaluated to determine whether items within grouping are, in reality, subtle variations of the same question. Item groupings with alpha < 0.4 will be rejected. Several criteria will be applied in evaluation of dimensions and factor retention: the Kaiser-Guttman rule (Eigenvalues > 1.0), factor loading thresholds of 0.60/0.40, scree plots, Kaiser–Meyer–Olkin (KMO) residuals off-diagonal partial correlations measure of sampling adequacy > 0.70 [28, 39, 40]. This analysis will be iterative. Emergent factor structures across mode and sample will be compared with the aim of identifying factor structures that are consistent across mode and across sample. The postpartum sample data will be evaluated similarly, with comparison of the factor structure made to final factor structure from general population sample and general clinical sample.

Our evaluation of levels of evidence is based on the work of Campbell and Fiske’s Multitrait-Multimethod (MTMM) matrix relative to the use of multiple-operationalism (multiple items to assess the range of a concept) to evaluate and establish levels of validity, although we use the term “dimension” in lieu of “trait.” [31]. Correlation values across multiple data sources and methods within each emergent dimension (factor) populating the MTMM matrix will allow evaluation of convergent validity and divergent validity.

Sample sizes are based on ensuring sufficient numbers of item response to conduct factor analysis. We aim to enroll the number of women necessary to achieve 10 participants per PLUS-BHI item included in the dimensional analysis [41], although less conservative estimates of 5–10 participants per item have been suggested [42]. Based on a maximum of 90 items a participant may respond to, our target number of completed BHIs in the general population sample (n = 694) and the clinical sample (n = 354) provides 8 and up to 12 participants per item evaluated, dependent on merging samples. This estimate is also conservative in that it accounts for all branching and not all participants will indeed branch based on their responses.

## Discussion

### Challenges in validating “health”

Many health care providers have spent their clinical and research careers studying and treating women with urinary symptoms as evidenced by the greater than 50 validated instruments to assess symptoms and quality of life impact in women with LUTS [43]. Previous efforts have focused on defining and treating the disease and not on prevention. The long-term goal of the PLUS Consortium is to decrease the incidence and prevalence of LUTS in women by identifying and then modifying risk and protective factors. Central to this goal is the ability to measure bladder health across a woman’s life course. The PLUS-BHI development and validation described in this manuscript is considered by the PLUS Consortium to be the cornerstone of future efforts in LUTS prevention research.

Critical to the development of the PLUS-BHS for research purposes is having a definition for bladder health [9, 44]. One of the greatest challenges of developing the PLUS-BHS is that we are measuring an abstract theoretical construct such as bladder sensation (a component of bladder health) by asking specific questions that are intended to capture the range of meaning of normal and abnormal “bladder sensation” [45]. We recognize that the questions about bladder function are likely to be interpreted in numerous ways by respondents due to the inability to discriminate between the responses. This is compounded by the reality that the presence of bladder symptoms in women are often considered a “normal” part of aging or secondary to childbirth. As a result, a woman might choose a response that describes her urinary leakage accurately, but attribute minimal disruption in her quality of lifestyle or bother to the symptom because she has been conditioned to believe it is normal. In addition, some LUTS are episodic such as stress incontinence or urinary tract infection symptoms yet are contributors or detractors from bladder health. Additional challenges to measuring overall bladder health relate to the fact that responses to questions can vary based on whether or not a woman is experiencing symptoms at the time of the instrument completion or has ever experienced any symptoms across her life course. Finally, it is challenging to design a measurement of health that is generalizable and interpretable across populations, the life course and settings. To address these issues, we use multiple samples across population and bladder health states and include a clinical interview and review of specific bladder testing by a variety of judges to improve use of the instrument across contexts.

### Strengths and limitations

One of the primary intended uses of the validated PLUS-BHS is to establish a U.S. general population estimate of women’s bladder health, across the life course. To ensure the PLUS-BHS is valid for this inference it is essential to validate using a national general population sample of women. Some of the challenges in conducting national population research is the identification of a representative sample frame, minimizing the well-known problem of coverage error. With survey methods sampling, the DSF with household level enumeration of every known mailing address within the U.S. is the address base sampling frame with the lowest coverage error that exists. The use of random assignment of both general population participants as well as, general clinical participants to the multiple modes (PAPI, CASI and within CASI Web primary as well as push to web) allows a valid and unconfounded comparison of differences in both response rate, item-response as well as distributional differences across mode. The inclusion of clinical samples using community based recruitment allows a clinical evaluation of women across the spectrum of potential bladder health. The inclusion of objective clinical test data provides the “hard ratings” required as a standard of evidence for valid inference with the intended use of the PLUS-BHS. A major strength of our validation work is the inclusion and evaluation of women during the postpartum period. While we would not expect women’s bladder health to be considered stable during pregnancy, we expect the postpartum period to be a factor in women’s bladder health over their lifetime.

While the goal of the PLUS-BHS is to be used across the female lifespan including adolescent girls and adult women, this initial validation was limited to English speaking women ≥ 18 years of age. The development of a similar instrument for adolescents and Spanish speaking females is underway and will be facilitated by the outcomes of the VIEW study. Since the instrument is specifically designed for women, who have different lower urinary tract anatomy and symptoms when compared to men or transgender women, this instrument is not intended for use with males or adolescent females. Lastly, the PLUS-BHI is only tested for clinical research in ambulatory women; thus, women who have functional and neurogenic LUTS are not included in the spectrum of bladder health assessed by the PLUS-BHI. The questions and answer stems might not be appropriate in this population and would need further testing to determine their ability to discriminate bladder health from bladder disease in certain populations.

The VIEW study includes women from the general population and from those recruited around clinical centers, those who are recently postpartum and who attend clinical practices for health care. While this range of recruitment can be a strength of the study to assure a wide range of contexts, it is also possible that the distribution of bladder health may not be comparable between the populations. While clinical center recruitment was intentionally designed in order to capture the full spectrum of very healthy to very unhealthy, the screening instrument we used (the PPBC) may not accurately discriminate across the true severity of disease and does not address impact of symptoms. Similarly, validated questionnaires were not used to stratify women according to type of LUTS for inclusion in the clinical examination. It is also possible that women recruited from clinical practices, specifically specialty practices (e.g. urology, urogynecology) and with self-reported severe symptoms may be fundamentally different than women who may have symptoms but who do not seek out care for LUTS. As a result, the PLUS-BHS may discriminate between participants with similar LUTS who have different expectations for their bladder health. Modifications to the scoring of the PLUS-BHS may need to be made for general, clinical and postpartum populations and the PLUS-BHS would not be as generalizable as desired.

The VIEW study participants are randomized to the use of paper or computer instruments in order to evaluate potential mode effect on items and subsequently develop a scale that is mode agnostic. While we aim to have a mode agnostic instrument, we acknowledge that it may not be feasible to conduct population based research using CASI mode given systemic biases associated with women’s access to technology. Additionally, while the use of smartphones and technology has increased in many parts of the U.S., we anticipate differences in response rates by mode of administration across age groups and by socioeconomic status.

In conclusion, we present the rationale and approach to development and validation of a novel instrument for the measurement of bladder health in epidemiologic research. The scored bladder health scale will provide the foundation to assess the distribution of bladder health in women and girls within the United States and allow for future study of a variety of factors associated with bladder health. With this foundational instrument developed, future iterations and adaptations will be possible to include adolescent and Spanish-speaking females and pregnant populations. Additional efforts in the future will focus on assessing sensitivity to change and minimum important differences for use in intervention studies. These data will inform future intervention trials in the promotion of bladder health and prevention of lower urinary tract symptoms in women across the life course.

## Supplementary information

**Additional file 1.** 2-Day Bladder Diary. Demonstrates 2 day voiding symptom and frequency diary used as criterion measure.

**Additional file 2.** 1-Day Bladder Diary. Demonstrates 24 h frequency volume diary used as criterion measure.

**Additional file 3.** Judge Rating Scale. Demonstrates data collection form used for judge ratings of bladder health.

## Data Availability

Data collected from this study will be reported in a separate publication and will be made available per NIH public use policies after the primary outcomes of the research are published.

## Abbreviations

BHI: Bladder Health Instrument
BFLUTS: Bristol Female Lower Urinary Tract
BHS: Bladder Health Scale
CASI: Computer Assisted Self-Administered Instrument
CRADI: Colorectal and anal distress inventory
DSF: Delivery Sequence File
KHQ: King’s Health Questionnaire
LUTS: Lower Urinary Tract Symptoms
MOS: Medical Outcome Study (MOS)
PAPI: Paper and Pencil Instrument
PFDI-20: Pelvic Floor Distress Inventory
PLUS: Prevention of Lower Urinary tract Symptoms
POPDI: Pelvic organ prolapse distress inventory
PPBC: Patient Perception of Bladder Condition
PPT: Paper Towel Test for stress incontinence
PSU: Primary Sampling Unit
PVR: Post-void residual volume
SAQ: Self-administered Questionnaire
SDCC: Scientific and Data Coordinating Center
SF-36: Short Form Health Survey
SSU: Secondary Sampling Unit
RA: Randomization Assignment
UDI: Urogenital Distress Inventory
VIEW: Validation of Bladder Health Instrument for Evaluation in Women
