# Supplementary material for: Rationale and design of the validation of bladder health instrument for evaluation in women (VIEW) protocol
Source: BMC Womens Health. 2021 Jan 7;21:18. doi: 10.1186/s12905-020-01136-w (PMC7789348; doi:10.1186/s12905-020-01136-w)
Supplement: Supplementary file 1 — Additional file 1. 2-Day Bladder Diary. Demonstrates 2 day voiding symptom and frequency diary used as criterion measure. [file 12905_2020_1136_MOESM1_ESM.pdf]

PREVENTION OF LOWER URINARY TRACT SYMPTOMS
A Research Consortium of the National Institutes of Health

2-DAY BLADDER HEALTH

SYMPTOM DIARY

PLEASE COMPLETE THIS DIARY FIRST

Participant ID:

Instructions for completing diary

Please complete the 2-Day Bladder Health Symptom Diary BEFORE completing the 1-Day Frequency-Volume Bladder Diary. For two days, we are asking you to record every time you pee or leak urine, as well as your experiences when peeing and after peeing. The two days you record on the 2-Day Symptom Diary should be done on two days in a row.

Choose any 2 days (48-hour period of time) to keep this Diary. You will need to take this Diary with you when you are at home, work or other locations to record your symptoms every time you pee (urinate).

TO COMPLETE THE DIARY:

Begin your Diary with the FIRST time you pee after you wake up from sleep.

Questions 1-8: Complete the questions about your health and your bladder.

At the Start of Each Day: Record the time you get up for the day.

COLUMN 1:

- Every time you pee or if you leak urine (even a drop), please check one of the boxes; P=Peed or L=Leaked. If you both leaked urine and peed, check the box marked "B" for Both.

COLUMN 2:

- Write down the time you peed in this column and check the box for AM or PM.

COLUMN 3:

- If you leaked pee, check if the amount was a small (S), medium (M), or large (L) leakage.

Column 4 – Column 6:

- Check Yes (Y) or No (N) for each question about any bladder urgency, your pee experience, and your after-pee experience.

At the End of Each Day:

- Check Yes (Y) or No (N) if you had an uncomfortable or painful pee sensation or if you experienced pain while holding urine.
- Answer whether this was a typical or normal day for you. If it was not, record why in the box.
- Record the time you go to bed.

EXAMPLE:

	Column 1	Column 2			Column 3			Column 4	Column 5		Column 6		
	Peed	Time of Pee or Leak			Accidental Leak			Urgency	Pee Experience		After-Pee Experience		
	Check Pee or Leak or Both	Time of Pee or Leak			Amount of Pee Leakage (check one if leak) Small (S)	Medium (M)	Large (L)	Had a sudden and urgent need to pee	Easy starting to pee	Continuous pee stream	Do you feel bladder is empty?	Is the "need to pee feeling" gone?	Did you dribble pee when you were done?
1	☐ P ☐ L ☒ B	5 : 35	☒ AM	☐ PM	☐ S	☒ M	☐ L	☐ Y ☒ N	☒ Y ☐ N	☒ Y ☐ N	☐ Y ☒ N	☐ Y ☒ N	☒ Y ☐ N

Please complete the following questions.

1. Please enter today's date: ☐☐ / ☐☐ / 2 0 ☐☐
   M M / D D / Y Y Y Y

2. In general, would you say your health is:
   - ☐ Excellent
   - ☐ Very good
   - ☐ Good
   - ☐ Fair
   - ☐ Poor

3. Are you breastfeeding?
   - ☐ Yes
   - ☐ No

4. Do you think you have a bladder infection or UTI today?
   - ☐ Yes
   - ☐ No

5. Are you pregnant?
   - ☐ Yes
   - ☐ No

6. Are you having any respiratory issues (such as a cold or allergies) today?
   - ☐ Yes
   - ☐ No

7. Are you catheterized?
   - ☐ Yes
   - ☐ No

8. Have you been hospitalized in the past week?
   - ☐ Yes
   - ☐ No

Participant ID: ☐

DAY 1

DAY 1

What time did you get up today? ☐ : ☐ ☐ AM ☐ PM

	Column 1	Column 2	Column 3			Column 4	Column 5		Column 6		
	Peed	Time of Pee or Leak	Accidental Leak or Lost Control of Pee			Urgency	Pee Experience		After-Pee Experience		
	Check **P**ee or **L**eak or **B**oth	Time of Pee or Leak	Amount of Pee Leakage (check one if leak, even just a drop or two) Small (S)	Medium (M)	Large (L)	A sudden and urgent need to pee, that "gotta go" feeling	Easy starting to pee	Continuous pee stream	Do you feel bladder is empty?	Is the "need to pee feeling" gone?	Did you dribble pee, even a few drops, when you were done?
1	☐ P ☐ L ☐ B	: ☐ AM ☐ PM	☐ S	☐ M	☐ L	☐ Y ☐ N	☐ Y ☐ N	☐ Y ☐ N	☐ Y ☐ N	☐ Y ☐ N	☐ Y ☐ N
2	☐ P ☐ L ☐ B	: ☐ AM ☐ PM	☐ S	☐ M	☐ L	☐ Y ☐ N	☐ Y ☐ N	☐ Y ☐ N	☐ Y ☐ N	☐ Y ☐ N	☐ Y ☐ N
3	☐ P ☐ L ☐ B	: ☐ AM ☐ PM	☐ S	☐ M	☐ L	☐ Y ☐ N	☐ Y ☐ N	☐ Y ☐ N	☐ Y ☐ N	☐ Y ☐ N	☐ Y ☐ N
4	☐ P ☐ L ☐ B	: ☐ AM ☐ PM	☐ S	☐ M	☐ L	☐ Y ☐ N	☐ Y ☐ N	☐ Y ☐ N	☐ Y ☐ N	☐ Y ☐ N	☐ Y ☐ N
5	☐ P ☐ L ☐ B	: ☐ AM ☐ PM	☐ S	☐ M	☐ L	☐ Y ☐ N	☐ Y ☐ N	☐ Y ☐ N	☐ Y ☐ N	☐ Y ☐ N	☐ Y ☐ N
6	☐ P ☐ L ☐ B	: ☐ AM ☐ PM	☐ S	☐ M	☐ L	☐ Y ☐ N	☐ Y ☐ N	☐ Y ☐ N	☐ Y ☐ N	☐ Y ☐ N	☐ Y ☐ N
7	☐ P ☐ L ☐ B	: ☐ AM ☐ PM	☐ S	☐ M	☐ L	☐ Y ☐ N	☐ Y ☐ N	☐ Y ☐ N	☐ Y ☐ N	☐ Y ☐ N	☐ Y ☐ N
8	☐ P ☐ L ☐ B	: ☐ AM ☐ PM	☐ S	☐ M	☐ L	☐ Y ☐ N	☐ Y ☐ N	☐ Y ☐ N	☐ Y ☐ N	☐ Y ☐ N	☐ Y ☐ N
9	☐ P ☐ L ☐ B	: ☐ AM ☐ PM	☐ S	☐ M	☐ L	☐ Y ☐ N	☐ Y ☐ N	☐ Y ☐ N	☐ Y ☐ N	☐ Y ☐ N	☐ Y ☐ N
10	☐ P ☐ L ☐ B	: ☐ AM ☐ PM	☐ S	☐ M	☐ L	☐ Y ☐ N	☐ Y ☐ N	☐ Y ☐ N	☐ Y ☐ N	☐ Y ☐ N	☐ Y ☐ N
11	☐ P ☐ L ☐ B	: ☐ AM ☐ PM	☐ S	☐ M	☐ L	☐ Y ☐ N	☐ Y ☐ N	☐ Y ☐ N	☐ Y ☐ N	☐ Y ☐ N	☐ Y ☐ N
12	☐ P ☐ L ☐ B	: ☐ AM ☐ PM	☐ S	☐ M	☐ L	☐ Y ☐ N	☐ Y ☐ N	☐ Y ☐ N	☐ Y ☐ N	☐ Y ☐ N	☐ Y ☐ N
13	☐ P ☐ L ☐ B	: ☐ AM ☐ PM	☐ S	☐ M	☐ L	☐ Y ☐ N	☐ Y ☐ N	☐ Y ☐ N	☐ Y ☐ N	☐ Y ☐ N	☐ Y ☐ N
14	☐ P ☐ L ☐ B	: ☐ AM ☐ PM	☐ S	☐ M	☐ L	☐ Y ☐ N	☐ Y ☐ N	☐ Y ☐ N	☐ Y ☐ N	☐ Y ☐ N	☐ Y ☐ N
15	☐ P ☐ L ☐ B	: ☐ AM ☐ PM	☐ S	☐ M	☐ L	☐ Y ☐ N	☐ Y ☐ N	☐ Y ☐ N	☐ Y ☐ N	☐ Y ☐ N	☐ Y ☐ N
16	☐ P ☐ L ☐ B	: ☐ AM ☐ PM	☐ S	☐ M	☐ L	☐ Y ☐ N	☐ Y ☐ N	☐ Y ☐ N	☐ Y ☐ N	☐ Y ☐ N	☐ Y ☐ N

Pee sensation uncomfortable or painful?

☐ Yes ☐ No

Did you experience pain while you were holding urine?

☐ Yes ☐ No

What time did you go to bed today? ☐ : ☐ ☐ AM ☐ PM

Participant ID: ☐

Did this represent a typical or normal day for you?

☐ Yes, normal

☐ No, worse → If no, please state what was different below:

☐ No, better → If no, please state what was different below:

☐

Participant ID: ☐

DAY 2

DAY 2

What time did you get up today? ☐ : ☐ ☐ AM ☐ PM

	Column 1	Column 2	Column 3			Column 4	Column 5		Column 6		
	Peed	Time of Pee or Leak	Accidental Leak or Lost Control of Pee			Urgency	Pee Experience		After-Pee Experience		
	Check Pee or **L**eak or **B**oth	Time of Pee or Leak	Amount of Pee Leakage (check one if leak, even just a drop or two): Small (S)	Medium (M)	Large (L)	A sudden and urgent need to pee, that "gotta go" feeling	Easy starting to pee	Continuous pee stream	Do you feel bladder is empty?	Is the "need to pee feeling" gone?	Did you dribble pee, even a few drops, when you were done?
17	☐ P ☐ L ☐ B	: ☐ AM ☐ PM	☐ S	☐ M	☐ L	☐ Y ☐ N	☐ Y ☐ N	☐ Y ☐ N	☐ Y ☐ N	☐ Y ☐ N	☐ Y ☐ N
18	☐ P ☐ L ☐ B	: ☐ AM ☐ PM	☐ S	☐ M	☐ L	☐ Y ☐ N	☐ Y ☐ N	☐ Y ☐ N	☐ Y ☐ N	☐ Y ☐ N	☐ Y ☐ N
19	☐ P ☐ L ☐ B	: ☐ AM ☐ PM	☐ S	☐ M	☐ L	☐ Y ☐ N	☐ Y ☐ N	☐ Y ☐ N	☐ Y ☐ N	☐ Y ☐ N	☐ Y ☐ N
20	☐ P ☐ L ☐ B	: ☐ AM ☐ PM	☐ S	☐ M	☐ L	☐ Y ☐ N	☐ Y ☐ N	☐ Y ☐ N	☐ Y ☐ N	☐ Y ☐ N	☐ Y ☐ N
21	☐ P ☐ L ☐ B	: ☐ AM ☐ PM	☐ S	☐ M	☐ L	☐ Y ☐ N	☐ Y ☐ N	☐ Y ☐ N	☐ Y ☐ N	☐ Y ☐ N	☐ Y ☐ N
22	☐ P ☐ L ☐ B	: ☐ AM ☐ PM	☐ S	☐ M	☐ L	☐ Y ☐ N	☐ Y ☐ N	☐ Y ☐ N	☐ Y ☐ N	☐ Y ☐ N	☐ Y ☐ N
23	☐ P ☐ L ☐ B	: ☐ AM ☐ PM	☐ S	☐ M	☐ L	☐ Y ☐ N	☐ Y ☐ N	☐ Y ☐ N	☐ Y ☐ N	☐ Y ☐ N	☐ Y ☐ N
24	☐ P ☐ L ☐ B	: ☐ AM ☐ PM	☐ S	☐ M	☐ L	☐ Y ☐ N	☐ Y ☐ N	☐ Y ☐ N	☐ Y ☐ N	☐ Y ☐ N	☐ Y ☐ N
25	☐ P ☐ L ☐ B	: ☐ AM ☐ PM	☐ S	☐ M	☐ L	☐ Y ☐ N	☐ Y ☐ N	☐ Y ☐ N	☐ Y ☐ N	☐ Y ☐ N	☐ Y ☐ N
26	☐ P ☐ L ☐ B	: ☐ AM ☐ PM	☐ S	☐ M	☐ L	☐ Y ☐ N	☐ Y ☐ N	☐ Y ☐ N	☐ Y ☐ N	☐ Y ☐ N	☐ Y ☐ N
27	☐ P ☐ L ☐ B	: ☐ AM ☐ PM	☐ S	☐ M	☐ L	☐ Y ☐ N	☐ Y ☐ N	☐ Y ☐ N	☐ Y ☐ N	☐ Y ☐ N	☐ Y ☐ N
28	☐ P ☐ L ☐ B	: ☐ AM ☐ PM	☐ S	☐ M	☐ L	☐ Y ☐ N	☐ Y ☐ N	☐ Y ☐ N	☐ Y ☐ N	☐ Y ☐ N	☐ Y ☐ N
29	☐ P ☐ L ☐ B	: ☐ AM ☐ PM	☐ S	☐ M	☐ L	☐ Y ☐ N	☐ Y ☐ N	☐ Y ☐ N	☐ Y ☐ N	☐ Y ☐ N	☐ Y ☐ N
30	☐ P ☐ L ☐ B	: ☐ AM ☐ PM	☐ S	☐ M	☐ L	☐ Y ☐ N	☐ Y ☐ N	☐ Y ☐ N	☐ Y ☐ N	☐ Y ☐ N	☐ Y ☐ N
31	☐ P ☐ L ☐ B	: ☐ AM ☐ PM	☐ S	☐ M	☐ L	☐ Y ☐ N	☐ Y ☐ N	☐ Y ☐ N	☐ Y ☐ N	☐ Y ☐ N	☐ Y ☐ N
32	☐ P ☐ L ☐ B	: ☐ AM ☐ PM	☐ S	☐ M	☐ L	☐ Y ☐ N	☐ Y ☐ N	☐ Y ☐ N	☐ Y ☐ N	☐ Y ☐ N	☐ Y ☐ N

Pee sensation uncomfortable or painful?

☐ Yes ☐ No

Did you experience pain while you were holding urine?

☐ Yes ☐ No

What time did you go to bed today? ☐ : ☐ ☐ AM ☐ PM

Participant ID: ☐

Did this represent a typical or normal day for you?

☐ Yes, normal

☐ No, worse → If no, please state what was different below:

☐ No, better → If no, please state what was different below:

☐

Participant ID: ☐
