# Supplementary material for: Rationale and design of the validation of bladder health instrument for evaluation in women (VIEW) protocol
Source: BMC Womens Health. 2021 Jan 7;21:18. doi: 10.1186/s12905-020-01136-w (PMC7789348; doi:10.1186/s12905-020-01136-w)
Supplement: Supplementary file 2 — Additional file 2. 1-Day Bladder Diary. Demonstrates 24 h frequency volume diary used as criterion measure. [file 12905_2020_1136_MOESM2_ESM.pdf]

1-DAY BLADDER HEALTH FREQUENCY-VOLUME DIARY

PLEASE COMPLETE THIS DIARY <u>AFTER</u> COMPLETING THE 2-DAY DIARY

Participant ID:

Instructions for completing diary

The 1-Day Bladder Health Frequency-Volume Diary can be completed any time AFTER you complete the 2-Day Symptom Diary. Choose any day of the week to start the 1-day Diary and keep track for 24 hours. Because you will need to measure your urine, you may want to complete the Diary when you are mostly at home.

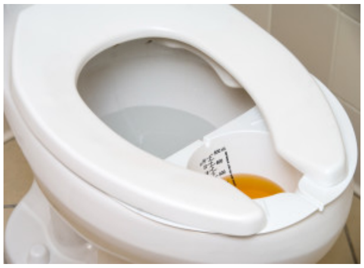

You will need to measure the amount you pee in milliliters (mL) using the plastic urine container you were given. Put the container in the toilet so the wings are under your toilet seat and the toilet seat will sit on top of the container. The straight part of the hat should be facing the back of the toilet. As shown in the picture, make sure the seat is down before you sit.

<u>TO COMPLETE THE DIARY:</u>

Begin your Diary with the FIRST time you pee after you wake up from sleep.

Question 1: Enter today's date.

Question 2 & 3: Record the time you get up for the day and the time you go to bed.

Question 4 & 5: Answer Yes or No for each question.

Question 6: During the 24 hours you are completing the diary, record all of the liquid you drink in ounces and enter the total.

Question 7: Answer whether this was a typical or normal day for you. If it was not, record why in the box.

Question 8: If you use pads for urine leakage or for protection, you should record the number and the type.

COLUMN 1:

- Every time you pee or if you leak urine (even a drop), please check one of the boxes; P=Peed or L=Leaked. If you both leaked urine and peed, check the box marked "B" for Both.
- Record the amount you peed in mL. After you write in the amount, you can empty the urine in the container in the toilet.

COLUMN 2:

- Write down the time you peed in this column and check the box for AM or PM.

COLUMN 3:

- If you leaked pee, check if the amount was a small (S), medium (M), or large (L) leakage.

EXAMPLE:

	Column 1		Column 2	Column 3		
	Peed		Time of Pee or Leak	Accidental Leak		
	Check Pee or Leak or Both	Volume Voided in mL	Time of Pee or Leak	Amount of Pee Leakage (check one if leak) Small (S)	Medium (M)	Large (L)
1	☐ P ☐ L ☒ B	250	5 : 35 ☒ AM ☐ PM	☐ S	☐ M	☒ L

Please complete the following questions.

1. Please enter today's date: ☐☐ / ☐☐ / 2 0 ☐☐

M M D D Y Y Y Y

2. What time did you get up today? ☐ : ☐ ☐ AM ☐ PM

3. What time did you go to bed today? ☐ : ☐ ☐ AM ☐ PM

4. Are you breastfeeding?

☐ Yes

☐ No

5. Do you think you have a bladder infection today?

☐ Yes

☐ No

6. How much fluid did you drink today? ☐ Ounces (8 ounces = 1 cup)

7. Did this represent a typical or normal day for you?

☐ Yes, normal

☐ No, worse → If no, please state what was different below:

☐ No, better → If no, please state what was different below:

☐

8. Did you use any pads for pee leaks?

☐ Yes → If yes, please list the number of pads used today:

☐ No

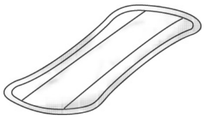

Pantyliners: ☐

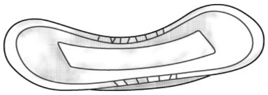

Maxi pads: ☐

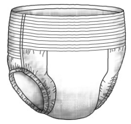

Pull-on/Adult Briefs with Tabs: ☐

Participant ID: ☐

	Column 1		Column 2					Column 3		
	Peed		Time of Pee or Leak					Accidental Leak		
	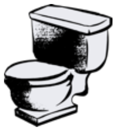 Check Pee or Leak or Both	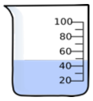 Volume Voided in mL	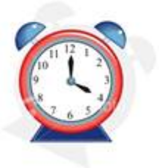 Time of Pee or Leak					Amount of Pee Leakage (check one if leak) Small (S) 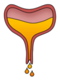	Medium (M) 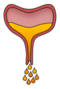	Large (L) 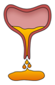
1	☐ P ☐ L ☐ B			:		☐ AM	☐ PM	☐ S	☐ M	☐ L
2	☐ P ☐ L ☐ B			:		☐ AM	☐ PM	☐ S	☐ M	☐ L
3	☐ P ☐ L ☐ B			:		☐ AM	☐ PM	☐ S	☐ M	☐ L
4	☐ P ☐ L ☐ B			:		☐ AM	☐ PM	☐ S	☐ M	☐ L
5	☐ P ☐ L ☐ B			:		☐ AM	☐ PM	☐ S	☐ M	☐ L
6	☐ P ☐ L ☐ B			:		☐ AM	☐ PM	☐ S	☐ M	☐ L
7	☐ P ☐ L ☐ B			:		☐ AM	☐ PM	☐ S	☐ M	☐ L
8	☐ P ☐ L ☐ B			:		☐ AM	☐ PM	☐ S	☐ M	☐ L
9	☐ P ☐ L ☐ B			:		☐ AM	☐ PM	☐ S	☐ M	☐ L
10	☐ P ☐ L ☐ B			:		☐ AM	☐ PM	☐ S	☐ M	☐ L
11	☐ P ☐ L ☐ B			:		☐ AM	☐ PM	☐ S	☐ M	☐ L
12	☐ P ☐ L ☐ B			:		☐ AM	☐ PM	☐ S	☐ M	☐ L
13	☐ P ☐ L ☐ B			:		☐ AM	☐ PM	☐ S	☐ M	☐ L
14	☐ P ☐ L ☐ B			:		☐ AM	☐ PM	☐ S	☐ M	☐ L
15	☐ P ☐ L ☐ B			:		☐ AM	☐ PM	☐ S	☐ M	☐ L
16	☐ P ☐ L ☐ B			:		☐ AM	☐ PM	☐ S	☐ M	☐ L
17	☐ P ☐ L ☐ B			:		☐ AM	☐ PM	☐ S	☐ M	☐ L
18	☐ P ☐ L ☐ B			:		☐ AM	☐ PM	☐ S	☐ M	☐ L
19	☐ P ☐ L ☐ B			:		☐ AM	☐ PM	☐ S	☐ M	☐ L
20	☐ P ☐ L ☐ B			:		☐ AM	☐ PM	☐ S	☐ M	☐ L
21	☐ P ☐ L ☐ B			:		☐ AM	☐ PM	☐ S	☐ M	☐ L
22	☐ P ☐ L ☐ B			:		☐ AM	☐ PM	☐ S	☐ M	☐ L
23	☐ P ☐ L ☐ B			:		☐ AM	☐ PM	☐ S	☐ M	☐ L
24	☐ P ☐ L ☐ B			:		☐ AM	☐ PM	☐ S	☐ M	☐ L
25	☐ P ☐ L ☐ B			:		☐ AM	☐ PM	☐ S	☐ M	☐ L

Participant ID: ____________
