# Supplementary material for: Rationale and design of the validation of bladder health instrument for evaluation in women (VIEW) protocol
Source: BMC Womens Health. 2021 Jan 7;21:18. doi: 10.1186/s12905-020-01136-w (PMC7789348; doi:10.1186/s12905-020-01136-w)
Supplement: Supplementary file 3 — Additional file 3. Judge Rating Scale. Demonstrates data collection form used for judge ratings of bladder health. [file 12905_2020_1136_MOESM3_ESM.pdf]

plus

FORM 5: JUDGE INITIAL RATING

Participant ID: __________ - ___ ___ ___

Judge ID: ____________________

Date: ___ ___ / ___ ___ / ___ ___ ___ ___
MM DD YYYY

Rating Based on Interview

Time you spent in interview: START TIME: _________ (24-hour clock) END TIME: _________ (24-hour clock)

Absolute Rating

1. How would you rate the health of this person's bladder? Please enter a rating from 0 – 10 in the textbox below.

10 — Best Bladder I Have Ever Seen
9
8
7
6
5 — About Average
4
3
2
1
0 — Worst Bladder I Have Ever Seen

Initial Rating: ___ ___ . ___

2. What are the three primary reasons for this rating?

I: ____
____
____
____

II: ____
____
____
____

III: ____
____
____
____

Adjusted/Relative Rating

1. Compared to other similarly situated women (e.g. age, health status, etc.), how would you rate the health of this person's bladder? Please enter a rating from 0 – 10 in the textbox below.

10 — Best Bladder I Have Ever Seen
9
8
7
6
5 — About Average
4
3
2
1
0 — Worst Bladder I Have Ever Seen

Adjusted Rating: ___ ___ . ___

2. What are the three primary reasons for this rating?

I: ____
____
____
____

II: ____
____
____
____

III: ____
____
____
____

Study Personnel Initials _______ Date Data Entered ___________

plus

FORM 6: JUDGE SECOND RATING

Participant ID: __________ - ___ ___ ___

Judge ID: ____________________

Date: ___ ___ / ___ ___ / ___ ___ ___ ___
MM DD YYYY

Rating Based on Interview & Additional Data

Did you access this participant's medical records?

☐ Yes ☐ No → Was this because you didn't want to or because you didn't have access to the medical records?

☐ Didn't want to access medical records ☐ Didn't have access to medical records

☐ I need additional information to complete this form → *Please complete form when information becomes available.*
For RCs: *Follow up with judge in 30 days. If judge can't make second assessment because of insufficient information, check here:* ☐

Absolute Rating

1. How would you rate the health of this person's bladder? Please enter a rating from 0 – 10 in the textbox below.

10 – Best Bladder I Have Ever Seen
9
8
7
6
5 – About Average
4
3
2
1
0 – Worst Bladder I Have Ever Seen

Initial Rating: ___ ___ . ___

2. What are the three primary reasons for this rating?

I: ______________________________

II: ______________________________

III: ______________________________

Adjusted/Relative Rating

1. Compared to other similarly situated women (e.g. age, health status, etc.), how would you rate the health of this person's bladder? Please enter a rating from 0 – 10 in the textbox below.

10 – Best Bladder I Have Ever Seen
9
8
7
6
5 – About Average
4
3
2
1
0 – Worst Bladder I Have Ever Seen

Adjusted Rating: ___ ___ . ___

2. What are the three primary reasons for this rating?

I: ______________________________

II: ______________________________

III: ______________________________
